# Determinant factors of gross domestic product (GDP) in Association of Southeast Asian Nations (ASEAN) member countries

**DOI:** 10.12688/f1000research.146826.1

**Published:** 2024-04-25

**Authors:** Faris Shafrullah, Leni Indrawati, Solahuddin Ismail, Faris Ihsan, Putri Ayu Pratiwi, Karno Karno

**Affiliations:** 1 Alumni University of Padjadjaran, Bandung, Indonesia; 2 University of Azzahra, Jakarta, Indonesia; 3 Universiti Utara Malaysia, Sintok, Kedah, Malaysia; 4 Main Expert BPSDMD, NTB Province, Indonesia; 5 Kupang State Polytechnic, Kupang, Indonesia; 6 University of Borobudur, Jakarta, Indonesia

**Keywords:** Gross Domestic Product (GDP), Foreign Direct Investment (FDI), foreign tourists, banking credit, inflation.

## Abstract

**Background:**

The gross domestic product (GDP) of Association of Southeast Asian Nations (ASEAN) member states can be used to determine their overall economic growth. In order to increase GDP, all emerging nations in Southeast Asia are stating to depend on foreign direct investment (FDI), the tourism industry, international visitors visiting ASEAN member nations banking credit, and low inflation rates. High inflation is a problem that developed and developing countries will definitely face. One of the problems faced by ASEAN member countries is that most of the member countries are in the developing country category. For ASEAN member countries, high inflation levels will encourage economic instability. GDP is less than optimal because of these classic problems in developing countries so that economic growth is not as planned. The ASEAN Economic Community (AEC), which ASEAN established, is seen as a way to reduce unemployment, alleviate poverty by boosting the travel and tourism industry and increasing investment, as well as to offer or distribute credit to businesses at low interest rates in order to boost GDP. The purpose of this study is to investigate and analyze how the GDP of ASEAN member nations is affected by FDI, foreign visitors, bank credit, and inflation. **s:** We analyzed panel data for seven ASEAN members from 2002 to 2020. FDI, foreign tourists, banking credit, and inflation are the research factors examined as independent variables that have an impact on GDP, the dependent variable.

**Results:**

The study’s findings indicate that while inflation has a large negative impact on GDP, FDI, foreign tourism, and bank credit have a considerable positive impact on GDP.

**Conclusions:**

FDI, foreign tourists, bank credit, and inflation have a significant influence on GDP in ASEAN member countries.

## Introduction

Economic growth is a process that, over time, raises the population’s real income per capita. It is also accompanied by institutional system modifications that have an impact on structural change and institutional transformation. Poverty, unemployment, low capital formation, sociocultural obstacles, corruption, and outside economic influence are all hurdles to economic progress ( Agarwal and Baron, 2023; Duican and Pop, 2015).

The importance of capital ownership is to create economic development in developing countries, but developing countries realize that the availability of capital is not yet a sufficient condition for creating economic development ( Asari *et al.*, 2011; Massidda and Etzo, 2012). Several other factors such as the availability of experts in various fields, sufficient entrepreneurs, an efficient government system, the ability to create and use more modern technology, and the attitude of society play an equally important role in creating economic development. Economic development problems are associated with economic growth ( Rasool *et al.*, 2021).

The expansion of economic activity results in a rise in the quantity of goods and services generated in society, which is known as economic growth ( Baltagi, 2005). Gross domestic product (GDP) is the market worth of all finished goods and services generated in a country within a certain time period ( Ganchev *et al.*, 2014; Belloumi, 2010). A high GDP value for a country is seen to suggest that the country has great economic health, whereas a low GDP value is thought to indicate weak economic health ( Demetriades and Hussein, 2006).

The entry of Foreign Direct Investment (FDI) into a country certainly has a significant influence on that country ( Mc Kinnon, 1964). Not only will capital come in, but technology and experts will also come in, which of course will have an impact on technology transfer and the country’s economic growth ( Katusiime, 2018; Borensztein *et al.*, 1998; Mankiw, 2009).

Foreign tourists are one aspect that can increase economic growth in ASEAN member countries. Visa exemptions between member countries also have an impact on increasing tourist visits ( Witt and Witt, 1992). Member countries are preparing and seriously building tourism industry infrastructure to serve visiting tourists by offering attractive tour packages. The tourism industry has a significant impact on economic growth ( Belloumi, 2010; Hymer *et al.*, 1976; Massidda and Etzo, 2012; Bouzahzah and Menyari, 2013; Rasool *et al.*, 2021).

Economic growth in a country is influenced by the economic conditions of neighboring countries and the condition of the financial infrastructure or infrastructure owned by that country ( Kelly *et al.*, 2013). The existence of bank credit can support economic growth in countries that have economic health in the short and long term ( Korkmaz, 2015; Mallik and Chowdhury, 2001). The existence of credit provided by banks to finance the development of financial infrastructure and other economic infrastructure has an influence on economic growth ( Duican and Pop, 2015; Ananzeh and Eddien, 2016; Hang Le *et al.*, 2021).

There is a feedback loop that has a significant impact between inflation and economic growth, where moderate inflation can help economic growth, but faster economic growth will have an impact on inflation ( Mankiw, 2009; Mallik and Chowdhury, 2001). A moderate and stable inflation rate will encourage the development process and economic growth of a country ( Pradhan *et al.*, 2013; Massidda and Etzo, 2012). Moderate inflation increases profits for savers, increases investment, and accelerates a country’s economic growth ( Saaed, 2008; Hasanov, 2011).

Association of Southeast Asian Nations (ASEAN) member countries are developing countries and have many problems, such as low foreign and domestic investment, high inflation, low levels of foreign and domestic tourists coming and visiting ASEAN countries, low credit disbursement, and development that tends to be uneven ( Puah *et al.*, 2018). GDP has not been maximized due to various classic problems in developing countries, so that economic growth is not as planned. The GDP of ASEAN countries can be seen in the table below.

The Table 1 above shows GDP from ASEAN countries that experienced fluctuations in the period 2016–2020. As a developing country, Indonesia’s economic situation in 2016 experienced an improvement, when compared to other ASEAN countries. The government of each country must be able to increase its country’s Gross Domestic Product (GDP) in order to increase its national development strength. Apart from that, domestic production must also continue to be increased with the contribution of labor. As the country’s development increases, new jobs will be created, thereby absorbing more new workers and reducing existing unemployment.

**Table 1.** Gross domestic oroduct (GDP) of Association of Southeast Asian Nations (ASEAN) countries 2016–2020.

Year	*Gross domestic product* (GDP) Member countries of ASEAN						
	Indonesia	Thailand	Philippines	Singapore	Malaysia	Vietnam	Brunei Darrusalam
2016	931.88	413.36	318.63	318.76	301.25	205.27	11.40
2017	1015.62	456.36	328.48	343.33	319.11	223.78	12.12
2018	1042.27	506.61	346.84	375.98	358.71	245.21	13.56
2019	1119.09	544.26	376.82	374.38	364.68	261.92	13.46
2020	1058.42	501.79	361.49	339.99	336.66	271.15	12.01

Source: World Bank.

The following Table 2 shows the foreign direct investment (FDI) using net inflows (Balance of payments, current USD) from seven ASEAN member countries.

**Table 2.** Foreign direct investment (FDI) in Association of Southeast Asian Nations (ASEAN) countries 2016–2020.

Year	*Foreign direct investment* (FDI) Member countries of ASEAN						
	Indonesia	Vietnam	Philippines	Thailand	Malaysia	Singapore	Brunei Darrusalam
2016	4.54	12.60	8.27	0.35	1.35	0.68	-0.15
2017	20.51	14.10	10.25	0.83	9.36	0.10	0.47
2018	18.91	15.50	9.94	1.32	8.30	8.31	0.51
2019	24.99	16.12	8.67	4.82	9.15	1.20	0.37
2020	18.58	15.80	6.54	4.80	3.51	0.90	0.57

Source: World Bank.

As seen in Table 2, FDI from ASEAN member countries in the period from 2016–2020 experienced fluctuations. During 2016–2019, the country most in demand for providing FDI was Singapore, because this country is the center of world trade (exports and imports), international financial services, and transportation services for world services and can be said to be ranked first ( Takahashi, 2020); however, in 2020, FDI experienced a drastic decline. FDI in Vietnam is ranked second and Indonesia is ranked third. Meanwhile, fourth place is occupied by Malaysia, the Philippines and Thailand ( Vicente *et al.*, 2017). Specifically, FDI in Brunei Darussalam increased during 2019–2020.

An overview of foreign tourists using international tourism, receipts (% of total exports) from seven ASEAN member countries can be seen below.

As seen in Table 3, Indonesia and the Philippines experienced an increase in foreign tourists from 2016 to 2019. In Vietnam, the number of foreign tourists decreased from 2016 to 2017 and remained stable from 2017 to 2018. In the countries of Malaysia, Singapore, Brunei Darussalam, and Thailand, the number of foreign tourists fluctuated from 2016 to 2020. The percentage of foreign tourists dropped significantly in ASEAN member countries in 2020.

**Table 3.** Foreign tourists to Association of Southeast Asian Nations (ASEAN) countries 2016–2020.

Year	Foreign tourists to ASEAN countries (%)						
	Indonesia	Malaysia	Philippines	Singapore	Thailand	Brunei Darussalam	Vietnam
2016	7.49	9.79	8.51	3.61	17.48	2.70	4.50
2017	7.56	9.08	9.64	3.39	18.73	2.94	3.90
2018	8.45	8.86	10.75	3.07	18.68	2.70	3.90
2019	9.20	9.33	12.12	3.30	20.09	19.80	4.21
2020	1.98	1.63	3.46	-1.03	5.94	9.70	1.11

Source: World Bank, CEIC Data, https://www.cnbcindonesia.com/, International Labor Organization (ILO), Economic Research Institute for ASEAN and East Asia, https://www.government.nl/, dan Philippine Statistic Authority.

Bank credit from seven ASEAN member countries using the domestic credit to private sector (% of GDP) indicator can be seen in the Table 4 below.

**Table 4.** Banking credit in Association of Southeast Asian Nations (ASEAN) countries 2016–2020.

Year	Banking credit in ASEAN member countries (%)						
	Indonesia	Malaysia	Filipina	Singapura	Thailand	Brunei Darussalam	Vietnam
2016	39.40	121.98	42.86	123.83	146.22	44.30	123.81
2017	38.73	117.17	45.61	120.96	144.64	39.46	130.72
2018	38.81	120.28	47.56	118.07	144.13	35.04	133.14
2019	37.75	120.67	47.97	120.03	143.29	35.71	137.91
2020	38.70	133.99	52.07	132.68	159.78	39.69	147.67

In Table 4, banking credit in Indonesia, Malaysia, Singapore, Thailand and Brunei Darussalam experienced fluctuations during the period 2016–2020. In 2020, the highest banking credit position was owned by Thailand and the lowest banking credit position was owned by Indonesia.

The fourth determining factor of GDP is inflation. The term “inflation” refers to an increase in commodity prices generally as a result of a misalignment between commodity procurement plans, production, price setting, money printing, and the amount of income held by the population. If the price of just one or two items rises, it is not considered inflation unless the increase spreads to other items or drives up the cost of the majority of other items ( Roy and Rahman, 2014). The issue is that prices have a propensity to rise consistently. In addition, price rises that happen seasonally, just before religious festivals, or that happen once and have no lasting effects are not considered inflation.

The inflation rate of seven ASEAN member countries that use consumer prices (annual %) as an indicator can be seen in Table 5 below.

**Table 5.** Inflation rates in Association of Southeast Asian Nations (ASEAN) countries 2016–2020.

Year	Inflation rate in ASEAN member countries (%)						
	Indonesia	Malaysia	Philippines	Singapore	Thailand	Brunei Darussalam	Vietnam
2016	3.53	2.09	1.25	-0.53	0.19	-0.28	2.67
2017	3.81	3.87	2.85	0.58	0.67	-1.26	3.52
2018	3.20	0.89	5.21	0.44	1.06	1.03	3.54
2019	3.03	0.66	2.48	0.57	0.71	-0.39	2.80
2020	1.92	-1.14	2.64	-0.18	-0.85	1.94	3.22

In Table 5, inflation in ASEAN member countries tends to fluctuate during the period 2016–2020. Overall, the inflation category experienced by ASEAN member countries is mild inflation. Mild inflation is inflation that has not yet disrupted the economic situation. This inflation can be controlled because prices are rising in general, but has not yet resulted in a crisis in the economic sector. Light inflation is below 10% per year.

### Research purposes

The purpose of the research is to examine and analyze the influence of FDI, foreign tourists visiting ASEAN member countries, banking credit, and inflation simultaneously on GDP in each ASEAN member country.

**Figure 1.**
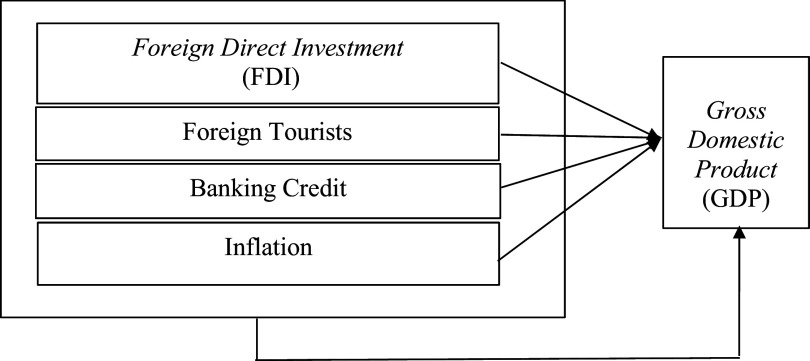
Framework.

The framework of thought can be seen in Figure 1 below:

The following is the research model:

GDPit=α+lnFDIit+TOURit+lnCRDit+INFLit+μit

Note:

GDP = Gross Domestic Product

lnFDI = Logaritma Natural Foreign Direct Investment

TOUR = Foreign Tourists

lnCRD = Logaritma Natural Bank Credit

INFL = Inflation

μ = error

I = Observation

T = Time

### Hypothesis

Foreign Direct Investment (FDI), foreign tourists, bank credit, and inflation influence Gross Domestic Product (GDP) in ASEAN member countries.

The increase or decrease in a country’s Gross Domestic Product (GDP) depends on several factors, including the flow of foreign investment, the number of visits by foreign tourists, the amount of credit provided by banks to entrepreneurs, and the level of inflation in the country. Therefore, the following hypotheses are formulated:

1. Foreign Direct Investment (FDI) has a significant effect on Gross Domestic Product (GDP) in ASEAN member countries.
2. Foreign tourists have a significant influence on Gross Domestic Product (GDP) in ASEAN member countries.
3. Bank credit has a significant effect on Gross Domestic Product (GDP) in ASEAN member countries.
4. Inflation has a significant effect on Gross Domestic Product (GDP) in ASEAN member countries.

## Methods

In total, seven ASEAN member countries were studied, namely Brunei Darussalam, Indonesia, Malaysia, the Philippines, Singapore, Thailand, and Vietnam. The sample data period covers 19 years, from 2002 to 2020, resulting in a total of 133 samples ( Karno *et al.*, 2023). The type of data used is panel data, and the analysis tool used is STATA Version 14.0. Data collection involved tracing sources from websites related to the variables studied. Below is Table 6, which presents a description of the variables, definitions, indicators, scale, and data sources used.

**Table 6.** Variables, Definitions, Indicators, Scale and Data Sources.

Variable	Definition	Indicator	Scale	Data source
Gross Domestic Product (GDP)	The value of goods and services produced within a country in a particular year	GDP (current US $)	Rasio	World Bank ( https://data.worldbank.org/)
Foreign Direct Investment (FDI)	Investments made with the aim of actively controlling property, assets or companies located in the host country	Foreign direct investment, net inflows (BoP, current US$)	Rasio	World Bank ( https://data.worldbank.org/) https://www.econstats.com/wdi/wdiv__42.htm Statista. ( https://www.statista.com/)
Foreign Tourists	A person who goes on a tourist trip enters a country other than the country where he usually lives	International tourism, receipts (% of total exports)	Rasio	World Bank ( https://data.worldbank.org/) CEIC Data ( https://www.ceicdata.com/id) International Labor Organization/ILO ( https://www.ilo.org) Economic Research Institute for ASEAN and East Asia ( https://www.eria.org/) https://www.government.nl/ Philippine Statistic Authority ( https://psa.gov.ph/) https://www.cnbcindonesia.com/ https://ekonomi.bisnis.com/
Banking Credit	The provision of money or the equivalent is based on a loan agreement between the bank and the borrower	Domestic credit to private sector (% of GDP)	Rasio	World Bank ( https://data.worldbank.org/) Econ Stats ( http://www.econstats.com/wdi/wdiv_440.htm)
Inflation	The trend of increasing prices of goods and services in general and continuously	Inflation, consumer prices (annual %)	Rasio	World Bank ( https://data.worldbank.org/) International Monetary Fund ( https://www.imf.org/)

## Results

The results of panel data regression using the Ordinary Least Squares, Fixed Effects, and Random Effects method are presented below.

The table above was carried out with the aim of comparing the regression results in three ways. It can be seen from the regression results in the OLS column above that the FDI and foreign tourist variables have a significant positive influence on GDP in each ASEAN member country ( Table 7). The banking credit variable has a significant negative influence on GDP in each ASEAN member country.

**Table 7.** Regression Test Results, OLS, Fixed Effects, Random Effects.

VARIABLE	OLS			FIXED EFFECTS			RANDOM EFFECTS		
	COEF	t	P >|t|	COEF	t	P >|t|	COEF	z	P >|z|
lnFDI	1.20	11.54	0.000	7.66	7.09	0.000	8.06	7,42	0.000
TOUR	1.92	5.22	0.000	1.56	3.39	0.001	1.56	3,40	0.001
lnCRD	-2.50	-8.57	0.000	1.57	3.07	0.003	1.06	2.14	0.032
INFL	-7.69	-1.84	0.069	-1.29	-4.03	0.000	-1.33	-4.08	0.000
Obs	130			130			130		
R ^2^	0.569			0.558			0.555		
Adj R ^2^	0.555								
Prob > F	0.000			0.000					
Prob > χ ^2^							0.000		

Source: Authors’ calculation using STATA 14.

The regression results in the Fixed Effect column above show that the variables FDI, foreign tourists and banking credit have a significant positive influence on GDP in each ASEAN member country. The inflation variable has a significant negative influence on GDP in each ASEAN member country ( Tamilselvan and Manikandan, 2015).

The regression results in the Random Effects column above show that the variables FDI, foreign tourists and banking credit have a significant positive influence on GDP in each ASEAN member country. The inflation variable has a significant negative influence on GDP in each ASEAN member country. If we compare the three test results, in the OLS method, the Inflation variable is not significant above 5%, while in the test results using the Fixed Effects and Random Effects methods all variables are significant below 5%. Next, the Fixed Effects and Random Effects methods were tested again to determine the best regression model using the Hausman Test method. Below are presented the results of the Hausman test.

From the regression results above, the variables FDI, foreign tourists and banking credit have a significant positive influence on GDP in each ASEAN member country. The inflation variable has a significant negative influence on GDP in each ASEAN member country.

From the description of the regression results using the Fixed Effects and Random Effects methods, the Hausman test was carried out to determine the best model. Below are presented the results of the Hausman test ( Table 8).

**Table 8.** Hausman test.

Variable	Coefficients		(b-B)	
	(b)	(B)		Prob > χ ^2^
	FE	RE	Difference	
lnFDI	7.66	8.06	-3.97	0.0012
TOUR	1.56	1.56	-5.96	
lnCRD	1.57	1.06	5.18	
INFL	-1.29	-1.33	3.61	

Source: Authors’ calculation using STATA 14.

Based on the table above, the results of the Hausman test between Fixed Effect and Random Effect show that the value in the Prob column (0.0012) is less than the critical value of 0.05 (chi 2 = 0.05). Therefore, the Alternative Hypothesis is accepted, namely Fixed Effect. If the value in the Prob > chi 2 column is significantly greater than 0.05, then the null hypothesis is accepted, or Random Effects is accepted. Therefore, the best model to be chosen is Fixed Effects.

## Discussion

**The results of the data analysis confirm the hypothesis that all variables have** a significant influence on the GDP variable in ASEAN member countries. Specifically:

1. Foreign Direct Investment (FDI) has a positive and significant effect on Gross Domestic Product (GDP) ( Borensztein *et al.*, 1998; Yazdi *et al*., 2017; Scarlett, 2021).
2. Foreign tourist arrivals have a positive and significant influence on GDP ( Belloumi, 2010; Bouzahzah and Menyari, 2013; Ohlan, 2017; Yazdi *et al*., 2017; Rasool *et al*., 2021; Scarlett, 2021).
3. Banking credit has a positive and significant effect on GDP ( Hang Le *et al.*, 2021; Duican and Pop, 2015; Ananzeh and Eddien, 2016).
4. Inflation has a significant negative effect on GDP ( Saaed, 2008; Hasanov, 2011; Obradovic *et al.*, 2017).

A detailed discussion of each variable’s impact on the GDP variable is provided below.

### GDP and FDI influence

Investments made with the intent to actively control real estate, other assets, or businesses based in a host nation are referred to as FDI. FDI inflows to developing countries in the Southeast Asia region are mainly carried out by investor countries seeking wider market potential. Large investments are mainly concentrated in advanced financial and technological industries. The presence of FDI as a driver of GDP in every developing country brings new capital, new technology and new skills.

The presence of FDI in the form of physical capital, advanced technology and professional experts brings benefits in the form of being able to process natural resources, increased employment opportunities, increasing sources of state revenue from taxes, and transfer of technology, management skills, and entrepreneurship. By attracting FDI, which is made possible by the cheap production costs in these emerging nations, the Southeast Asian region’s developing nations help their economies grow their GDP. Additionally, FDI provides these developing nations with more cutting-edge technology, allowing local businesses to imitate foreign businesses’ use of technology and local businesses to acquire that technology through labor mobility. In turn, developing nations enable developed nations to manage resources. nature in the form of a mutually beneficial connection. FDI inflows to ASEAN member nations are still very modest. The inflow of money in the form of FDI is anticipated to promote sustainable investment growth in ASEAN member nations because it is a form of long-term capital flow that is considerably less susceptible to economic unrest ( Borensztein *et al.*, 1998; Yazdi *et al*., 2017; Scarlett, 2021).

### The influence of foreign tourists on GDP

Foreigners who travel abroad on vacation and enter a country other than their home country are referred to as foreign tourists. The strategies used to attract foreign tourists are to develop priority destinations that become tourist attractions, revitalization of cultural heritage buildings that attract tourists, develop a tourism promotion cooperation network at local, national and even international levels with local and national media to introduce tourist attractions, developing the quality of tourist attractions to meet the desires and challenges of tourists, increasing the number of tourism destinations so that tourists have several choices for traveling, and developing tourism facilities and infrastructure. The tourism sub-sector contributes to the country’s foreign exchange earnings which can increase GDP through foreign tourist visits. Visits from foreign tourists have increased over time to ASEAN member countries which can increase the income of these ASEAN member countries, for example the country’s foreign exchange income generated through foreign tourist expenditure. Expenditures of foreign tourists are goods and services purchased by tourists in order to fulfill their needs, desires and hopes while they stay in the tourist destination areas they visit. Tourist spending in a country needs to be calculated carefully so that it can be known how much foreign exchange is earned in a particular country. Tourist expenses usually include hotel accommodation, bars and restaurants, local transportation, tours or sightseeing, souvenirs, and other necessities. This expenditure by foreign tourists can be said to be revenue from the tourism sector for a country visited by foreign tourists. Apart from that, the tourism sub-sector which influences other sectors is tour and travel which includes hotels, restaurants and tour guides, so it can be said that the tourism sub-sector is a dynamic sub-sector and influences other economic segments ( Belloumi, 2010; Bouzahzah and Menyari, 2013; Ohlan, 2017; Yazdi *et al*., 2017; Rasool *et al*., 2021; Scarlett, 2021).

### GDP and the impact of bank credit

According to a loan arrangement between the bank and the borrower, bank credit is the provision of money or something comparable. Since the banking sector acts as an intermediary and is one of the elements that influences economic movements in all sectors, including the industrial sector in this instance, the government distributes banking credit through banks, the banking sector’s contribution to raising GDP cannot be overlooked. The financial sector is believed to be able to increase GDP by diverting financial funds from unproductive to productive use. Banking credit can be grouped into two types, namely productive credit and non-productive credit. Productive credit has a positive contribution to GDP consisting of working capital credit and investment credit. While non-productive credit has a negative contribution to GDP, in this case it is consumption credit. Viewed from the demand and supply side of banking credit, for example housing credit, if the banking sector increases, the funds that can be allocated for housing credit will also increase. This will have an effect on GDP. The greater the funds collected from the public, the greater the amount of banking credit provided. Increasing the amount of bank credit disbursed will increase productivity so that GDP will be higher.

In ASEAN members Malaysia, Singapore, Thailand, and Vietnam, the banking industry beats Indonesia in terms of expanding GDP. Because several manufacturing industries in Indonesia have obtained bank credit but are having trouble operating and even experiencing bad credit, the government offers banking credit through banks or financial institutions, not only providing loans in the form of funds but also management development. Real economic activity is not always positively impacted by bank loans. The economy may be harmed by excessive bank credit, particularly private credit. In other words, once reaching the limit, the financial sector’s growth will no longer serve as a catalyst for development but rather a barrier to it ( Hang Le, *et al.*, 2021; Duican and Pop, 2015; Ananzeh and Eddien, 2016).

### The effect of inflation on GDP

Inflation is a general and continuous increase in the prices of goods. Inflation is an important economic indicator, where the growth rate is always kept low and stable so as not to cause economic instability ( Wooldridge and Jefferey, 2010). Light inflation has no effect on GDP, because light inflation does not cause prices to rise, so the amount of goods produced remains constant and causes GDP to remain unchanged or unchanged. On the other hand, moderate inflation, heavy inflation and hyperinflation have a negative impact on GDP. High GDP, low and stable inflation are the ideal targets that ASEAN member countries want to achieve. High inflation will push prices to become more expensive, which will cause companies to incur higher production costs. High production costs force companies to reduce direct purchases of raw materials and use of direct labor, which will also have an impact on reduced production of goods, so that people’s income will fall. The decline in people’s income causes a decline in people’s purchasing power for goods, where companies reduce the production of goods, which causes GDP to fall ( Saaed, 2008; Hasanov, 2011; Obradovic, *et al.*, 2017).

## Conclusion

Based on the results of the research and discussion, conclusions can be drawn, namely that FDI, foreign tourists, banking credit, and inflation have a significant effects simultaneously on GDP in ASEAN member countries. High FDI flows in the form of physical capital, technology and skilled workers to ASEAN member countries can increase GDP. The strategy carried out by countries in the Southeast Asia region is to increase tourism competitiveness through developing aspects of attractions, accessibility and amenities so that foreign tourist visits can increase GDP in each ASEAN member country. In increasing GDP in ASEAN member countries, the banking sector as an intermediary can trigger economic movements in all sectors including the industrial sector, in this case, the government provides banking credit through banks. ASEAN member countries would want to achieve low inflation so that GDP does not fall.

## Data taken from several sources, namely:

*World Bank* ( https://data.worldbank.org/)

CEIC Data ( https://www.ceicdata.com/id)

*United Nation Data/UN Data* ( http://data.un.org/)

Statista ( https://www.statista.com/)

*International Monetary Fund* ( https://www.imf.org/)

Indexmundi ( https://www.indexmundi.com/)

Knoema ( https://knoema.com/)

*International Labor Organization*/ILO ( https://www.ilo.org)

Filipina: https://psa.gov.ph/ (Philippine Statistic Authority)

Myanmar: https://tourism.gov.mm/statistics/ (Myanmar Tourism Statistic)

Singapura: https://www.mas.gov.sg/statistics (Monetary Authority of Singapore)

https://countryeconomy.com/

https://www.theglobaleconomy.com/

Economic Research Institute for ASEAN and East Asia ( https://www.eria.org/)

https://www.government.nl/

## Data Availability

Figshare: Underlying Data.xlsx. https://doi.org/10.6084/m9.figshare.24882699.v1 ( Karno *et al.*, 2023). The project contains the following underlying data:
- Underlying Data.xlsx Data are available under the terms of the Creative Commons Attribution 4.0 International license (CC-BY 4.0).
