# Supplementary material for: Determinant factors of gross domestic product (GDP) in Association of Southeast Asian Nations (ASEAN) member countries
Source: F1000Res. 2024 Sep 30;13:384. Originally published 2024 Apr 25. [Version 2] doi: 10.12688/f1000research.146826.2 (PMC11883207; doi:10.12688/f1000research.146826.2)
Supplement: Peer review history

# Reviewer report: Reviewer response for version 2

**DOI:** 10.5256/f1000research.172560.r365497

**Published:** 2025-03-05

**Authors:** Justice Gameli Djokoto

**Affiliations:** 1 Dominion University College, Central University, Accra, Ghana

The paper identified the determinants of GDP in ASEAN countries. 

 The background is adequate with the problem laid out. However, the literature gap that should lead to the contribution to the literature is unclear. This must be addressed in a revision. The modelling is adequate. The results are related to the empirical literature.

 The conclusions arise from the results.

Is the work clearly and accurately presented and does it cite the current literature?

Yes

If applicable, is the statistical analysis and its interpretation appropriate?

Yes

Are all the source data underlying the results available to ensure full reproducibility?

Yes

Is the study design appropriate and is the work technically sound?

Yes

Are the conclusions drawn adequately supported by the results?

Yes

Are sufficient details of methods and analysis provided to allow replication by others?

Yes

Reviewer Expertise:

Intenational economics

I confirm that I have read this submission and believe that I have an appropriate level of expertise to confirm that it is of an acceptable scientific standard.

# Reviewer report: Reviewer response for version 2

**DOI:** 10.5256/f1000research.172560.r367064

**Published:** 2025-02-27

**Authors:** Emre Gökçeli

**Affiliations:** 1 Kütahya Dumlupınar Üniversitesi, Kütahya, Turkey

First of all, thank you for the study. I am writing down the issues I have noticed regarding the study.

1. In the introduction section, there is no clear connection between the paragraphs. The factors affecting economic development have been briefly mentioned, but they have not been elaborated on.
2. More importantly, I have serious concerns about the reliability of the method used. The cross-sectional dependence (CSD) test has not been examined. If CSD is present, the OLS and Fixed Effects methods do not yield accurate results. Moreover, some pretests, such as the unit root test and correlation between variables, have been overlooked, which also leads to unreliable results.

Due to these serious issues, I believe that this manuscript does not meet the quality standards required for acceptance in Scopus.

Is the work clearly and accurately presented and does it cite the current literature?

Partly

If applicable, is the statistical analysis and its interpretation appropriate?

No

Are all the source data underlying the results available to ensure full reproducibility?

No

Is the study design appropriate and is the work technically sound?

Partly

Are the conclusions drawn adequately supported by the results?

Partly

Are sufficient details of methods and analysis provided to allow replication by others?

No

Reviewer Expertise:

Foreign Direct Investment, Economic Growth, Environmental Quality, Reenwable Energy,

I confirm that I have read this submission and believe that I have an appropriate level of expertise to state that I do not consider it to be of an acceptable scientific standard, for reasons outlined above.

# Reviewer report: Reviewer response for version 2

**DOI:** 10.5256/f1000research.172560.r328015

**Published:** 2024-11-15

**Authors:** Elistia Elistia

**Affiliations:** 1 Universitas Esa Unggul, West Jakarta, Jakarta, Indonesia

1. Hypothesis

 Hypothesis sentences must be consistent, using the words influence or effect. Then the sub-headings in each discussion are also consistent.

 2. Methods

 The sample period analyzed is from 2016 to 2020, but in the written method it starts from 2002 to 2020, then there are 133 samples, what sample is this, because the data processed and analyzed is time series data, the number of which is no more than 133, and the time series starts from 2016 - 2020. This must be corrected.

Is the work clearly and accurately presented and does it cite the current literature?

Partly

If applicable, is the statistical analysis and its interpretation appropriate?

Partly

Are all the source data underlying the results available to ensure full reproducibility?

Yes

Is the study design appropriate and is the work technically sound?

Yes

Are the conclusions drawn adequately supported by the results?

Partly

Are sufficient details of methods and analysis provided to allow replication by others?

Yes

Reviewer Expertise:

Economics, Strategic Management, Human Resources Management, Entrepreneurship.

I confirm that I have read this submission and believe that I have an appropriate level of expertise to confirm that it is of an acceptable scientific standard, however I have significant reservations, as outlined above.

# Reviewer report: Reviewer response for version 2

**DOI:** 10.5256/f1000research.172560.r337678

**Published:** 2024-11-11

**Authors:** Ariodillah Hidayat

**Affiliations:** 1 Sriwijaya University, Palembang, Indonesia

**Determinant factors of gross domestic product (GDP) in Association of Southeast Asian Nations (ASEAN) member countries.**

 This study is quite interesting because it examines factors that affect GDP such as inflation, foreign investment, tourism industry, foreign credit and tourists using ASEAN country panel data. However, there are a few things that need to be fixed:

1. In the abstract part, the researcher must highlight what the findings are and the implications of the findings. Too many sentences that don't need to be explained in the abstract. Simply the purpose of the analysis, the data period, the sample/population, the analysis technique and the conclusion of the suggestions.
2. In the introduction, it should not focus on data, but the phenomenon that is interested in research. In addition, the introduction is ineffective, the content is too long. There have been many studies with titles like this, but what is the latest and urgency of the research should be explained. If all variables have been studied before, researchers should be able to add new variables that can affect GDP.
3. Overall, especially in the introduction, there are still many citations under the last 10 years. It's a good idea to include recent references.
4. In the introduction, too many tables are displayed. It can be moved to the results section for further analysis of data trends.
5. The equation model in the study should be included in the methods subchapter.
6. The authors state that the best fixed effect model, in which the model allows for the result of a coefficient cross section, should also be included in the table and explained.
7. In the method section, it should be explained why 7 ASEAN countries are used.
8. In the discussion part of the first part, it should not be made in the form of points 1,2,3,4. It is better to just include it in the discussion between variables subchapter.
9. In the discussion between variables, do not repeat sentences such as definitions, definitions should be placed at the beginning of the introduction. In addition, in the subchapter on the impact of inflation on GDP, the author explains 10 ASEAN countries, while in the method section of 7 ASEAN countries studied. The author should analyze more specific policies that have been implemented in each of the 7 ASEAN countries to boost GDP, so that they can become best practices for policy. In addition, if quoting, it is better to put the quote after the quoted sentence, not at the end of all paragraphs.
10. The conclusions are too few, just repeating what was already in the previous section. It is better to give implications, what efforts are needed by ASEAN countries in increasing their GDP. Like the sentence: ASEAN member countries certainly want to achieve low inflation so that GDP does not fall. Such a sentence is only a statement, as a researcher should explain further how ASEAN countries are able to achieve the desired inflation, what efforts can be made.

Is the work clearly and accurately presented and does it cite the current literature?

Partly

If applicable, is the statistical analysis and its interpretation appropriate?

Yes

Are all the source data underlying the results available to ensure full reproducibility?

Yes

Is the study design appropriate and is the work technically sound?

Partly

Are the conclusions drawn adequately supported by the results?

Partly

Are sufficient details of methods and analysis provided to allow replication by others?

Yes

Reviewer Expertise:

Monetary, Industrial Economics, Microfinance, Fintech, AML CFT, Development Economics

I confirm that I have read this submission and believe that I have an appropriate level of expertise to confirm that it is of an acceptable scientific standard, however I have significant reservations, as outlined above.

# Reviewer report: Reviewer response for version 1

**DOI:** 10.5256/f1000research.160947.r270498

**Published:** 2024-09-26

**Authors:** Tariq Hussain

**Affiliations:** 1 Grand Asian University Sialkot, Sialkot, Punjab, Pakistan

- The title of the article “Determinant factors of gross domestic product (GDP) in Association of Southeast Asian Nations (ASEAN) member countries”, is sizzling and remained under the discussion of researchers and academic scholars since long.
- In my humble opinion the topic may be “Major Determinants of Gross Domestic Product (GDP) among Association of Southeast  Asian Nations (ASEAN) countries”.
- The write up of Abstract needs to improve regarding English language and methodology is not discussed in it. Please briefly introduce the methodology in the abstract
- Introduction is elaborated fully, however, the table presented in this part of the paper has time span 2016-2020, whereas the study time period is 2002-2020. Please justify this in paper
- In the intro, updated references are used but in less number
- Literature Review should be presented separately
- In the same way, Theoretical frame work is missing.
- Methodology is key part of any research paper, it is not fully elaborated. It is suggested to interpret in the paper
- Interpretation of empirical result is good. It would be better to discuss with the help of coefficient values and shows how many percentages change occur due to independent variables in dependent variables.
- Policy implications are based on the variables’ results, its highly appreciated.

Is the work clearly and accurately presented and does it cite the current literature?

Yes

If applicable, is the statistical analysis and its interpretation appropriate?

Yes

Are all the source data underlying the results available to ensure full reproducibility?

Yes

Is the study design appropriate and is the work technically sound?

Yes

Are the conclusions drawn adequately supported by the results?

Yes

Are sufficient details of methods and analysis provided to allow replication by others?

Partly

Reviewer Expertise:

My areas of research is Economic Growth Policies, Development Economics and Institutional Economics

I confirm that I have read this submission and believe that I have an appropriate level of expertise to confirm that it is of an acceptable scientific standard, however I have significant reservations, as outlined above.

# Reviewer report: Reviewer response for version 1

**DOI:** 10.5256/f1000research.160947.r293084

**Published:** 2024-08-29

**Authors:** Elistia Elistia

**Affiliations:** 1 Universitas Esa Unggul, West Jakarta, Jakarta, Indonesia

Determinant factors of gross domestic product (GDP) in Association of Southeast Asian Nations (ASEAN) member countries.

Abstract

 Provide brief implications and suggestions.

Introduction

 - Provide an explanation of the urgency and benefits of the research.

 - Present the relevant research gap.

 - Explain why the study focuses only on 7 ASEAN countries.

 - Provide an explanation of what constitutes a good inflation rate.

Research Purposes

 Figure 1. What framework?

Methods

 - The explanation of the research methods should examine each of the 7 ASEAN countries.

 - It is recommended that the data collected be up to the year 2022.

Results and discussion

 - Provide an explanation of how the policies of each of the 7 ASEAN countries relate to the variables of FDI, FT, BC, and Inf.

 - It is recommended to use recent references for the discussion explanation.

Conclusion

 - Mention only 7 countries, as the research only covers 7 ASEAN countries.

Is the work clearly and accurately presented and does it cite the current literature?

Partly

If applicable, is the statistical analysis and its interpretation appropriate?

Partly

Are all the source data underlying the results available to ensure full reproducibility?

Yes

Is the study design appropriate and is the work technically sound?

Yes

Are the conclusions drawn adequately supported by the results?

Partly

Are sufficient details of methods and analysis provided to allow replication by others?

Yes

Reviewer Expertise:

Economics, Strategic Management, Human Resources Management, Entrepreneurship.

I confirm that I have read this submission and believe that I have an appropriate level of expertise to confirm that it is of an acceptable scientific standard, however I have significant reservations, as outlined above.

## References

[1] : THE CORRELATION OF THE HUMAN DEVELOPMENT INDEX (HDI) TOWARDS ECONOMIC GROWTH (GDP PER CAPITA) IN 10 ASEAN MEMBER COUNTRIES. *JHSS (JOURNAL OF HUMANITIES AND SOCIAL STUDIES)* .2018;2(2) : 10.33751/jhss.v2i2.949 40-46 10.33751/jhss.v2i2.949

# Response

**Published:** 2024-09-26

**Authors:** Leni Indrawati

**Affiliations:** University of Azzahra, Jakarta, Indonesia

The revised article incorporates several key changes and updates based on the provided comments and suggestions. In the abstract, we have retained the original text, emphasizing the significant impact of FDI, foreign tourists, bank credit, and inflation on GDP in ASEAN countries, along with the suggestion for policymakers and stakeholders to prioritize relevant policies. The introduction now includes an explanation of what constitutes a good inflation rate, addressing a key aspect highlighted in the comments. Additionally, we have maintained the urgency and benefits of the research, presented the relevant research gap, and explained the focus on seven ASEAN countries. The methods section now includes a recommendation to collect data up to the year 2022, ensuring the analysis encompasses recent economic trends and developments, and provides a rationale for considering the period from 2002 to 2020. In the results and discussion section, we have incorporated an explanation of how the policies of each of the seven ASEAN countries relate to the variables of FDI, FT, BC, and Inf, as well as recent references to enhance the discussion. The conclusion now specifically mentions the seven countries covered in the research, and we have provided responses to the specific questions raised in the comments, addressing the clarity and accuracy of the work, appropriateness of the study design, technical soundness, provision of sufficient methodological details, appropriateness of statistical analysis and interpretation, availability of source data, and adequacy of conclusions in relation to the results. Overall, these changes have been made to address the comments thoroughly and improve the quality and relevance of the article.
